# Fibrolamellar Hepatocellular Carcinoma: Treatment with Yttrium-90 and Subsequent Surgical Resection

**DOI:** 10.1007/s00270-018-1903-6

**Published:** 2018-02-21

**Authors:** Sebastian Mafeld, Jeremy French, Dina Tiniakos, Beate Haugk, Derek Manas, Peter Littler

**Affiliations:** 1 Department of Interventional Radiology, Freeman Hospital, Newcastle upon Tyne, NE7 7DN UK; 2 Department of Hepatobiliary Surgery, Freeman Hospital, Newcastle upon Tyne, NE7 7DN UK; 3 Institute of Cellular Medicine, Faculty of Medical Sciences, Newcastle University, W. Leech Building, M4.143, Framlington Place, Newcastle upon Tyne, NE2 4HH UK; 4 Department of Cellular Pathology, Royal Victoria Infirmary, Newcastle upon Tyne Hospitals NHS Foundation Trust, Newcastle upon Tyne, NE1 4LP UK

**Keywords:** Fibrolamellar hepatocellular carcinoma, Radioembolization, SIRT, TARE, Yttrium-90, Liver resection

## Abstract

We describe a 52-year-old female patient who presented with a 9.5-cm fibrolamellar hepatocellular carcinoma (FL-HCC). The patient was initially unsuitable for surgical resection and therefore underwent transarterial chemoembolization followed by selective internal radiation therapy (SIRT) with Yttrium-90 to downsize the tumour. Following SIRT, the tumour decreased in volume from 350 to 20 cm^3^ allowing curative (R0) resection with an extended left hepatectomy and reconstruction of IVC. This is the first reported case of FL-HCC treated with SIRT in which, due to the good SIRT response, the patient was downsized to allow curative resection.

## Introduction

Fibrolamellar hepatocellular carcinoma (FL-HCC) is a rare variant of HCC. While the majority (70% of cases) present with resectable disease, for those who present with unresectable disease (20–30% of cases), there are limited treatment options with a 5-year survival between 0 and 5% and median survival of 12 months [1–3]. Those patients who are able to undergo resection have a 5-year survival approaching 70% [4]. FL-HCC is not typically responsive to chemotherapy, and while liver-directed therapies [transarterial chemoembolization (TACE) and ablation and selective internal radiation therapy (SIRT)] are evidence-based treatments for HCC, their role in FL-HCC is poorly understood [5–8].

Selective internal radiotherapy (SIRT) with Yttrium-90 (Y90) is an intra-arterial-directed therapy for hepatic malignancy. Small (20–60 microns) microspheres containing Y90, a beta-emitter, are infused into the target liver arteries in order to deliver a localized brachytherapy effect [9, 10]. As hepatic tumours are mostly supplied by the arterial system, the delivery of Y90-coated microspheres into the liver arteries is preferentially distributed in tumours, while aiming to spare normal liver parenchyma which is supplied by the portal venous system. SIRT is a well-described treatment for traditional HCC, but its use in FL-HCC has never been reported. We describe the successful delivery of SIRT and observe downsizing that facilitated curative surgical resection in a patient with biopsy-proven FL-HCC. Given the importance of resection in the management of FL-HCC, we hope this case adds to the literature in expanding treatment considerations in this difficult-to-manage patient group.

## Case

**Fig. 1:**
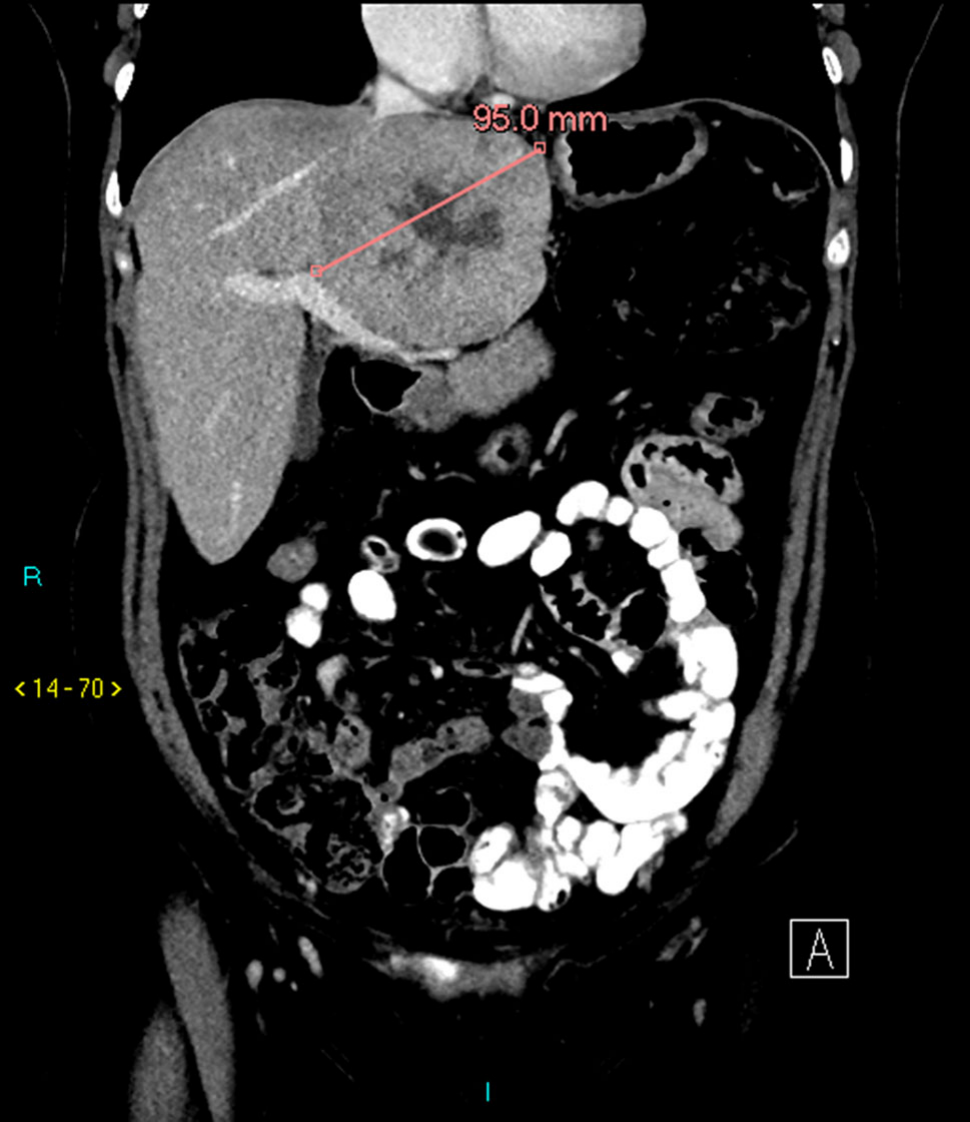
Coronal contrast-enhanced CT demonstrating a 9.5-cm (350 cm^3^) mass lesion centred in the caudate lobe but extending into segments 4a, 7 and 8

A 52-year-old female presented with a vague history of upper abdominal discomfort which prompted an ultrasound scan and subsequently demonstrated a large hepatic mass lesion. A CT and MRI were performed showing a 9.5-cm (350 cm^3^) mass lesion centred in the caudate lobe but extending into segments 4a, 7 and 8 (Fig. 1). Imaging features were non-diagnostic. Differential diagnoses included HCC or focal nodular hyperplasia. Alpha fetoprotein (AFP) (Roche method) was raised at > 50,000 IU/ML (normal range < 10 IU/ML). A laparoscopic biopsy was performed. Histological assessment revealed a FL-HCC with a background non-cirrhotic liver with only mild steatosis (Fig. 4). Due to the central location of the tumour and its involvement in the three hepatic veins at their insertion into the inferior vena cava (IVC), following multidisciplinary discussion the patient was deemed unresectable.

Treatment with liver-directed therapy in the form of TACE with doxorubicin-loaded drug-eluting beads was performed. At initial TACE, the FL-HCC was noted to have derived parasitic extrahepatic arterial supply from a phrenic branch arising off the proximal coeliac axis; this was treated with 75 mg of 100- to 300-micron doxorubicin-loaded beads (BTG, London, UK). Three weeks later the patient had a second TACE from branches of the left hepatic artery with a total dose of 150 mg of doxorubicin-loaded M1 (70–150 microns) drug-eluting beads (BTG, London, UK). Follow-up CT demonstrated a partial response on mRECIST criteria, but stable by RECIST. The tumour remained unresectable; therefore, following further multidisciplinary discussion, SIRT with Y90 was recommended to downsize the tumour. On the planning angiogram, the parasitic supply from the phrenic branch to the tumour was embolized and lung shunt fraction determined by injecting Technetium ^99m^Tc macro-aggregated albumin into the left and right hepatic arteries. Arterial supply to the tumour was confirmed by cone-beam CT images obtained from the right and left hepatic arteries (pre-SIRT procedure). A lung shunt fraction of 7% was calculated and the patient proceeded to staged (2-month interval) SIRT using TheraSpheres Y90 glass-coated microspheres with 3.45 GBq delivered to the right hepatic artery (15 months after initial TACE) and 1.18 GBq from the left hepatic artery (17 months after initial TACE).

**Fig. 2:**
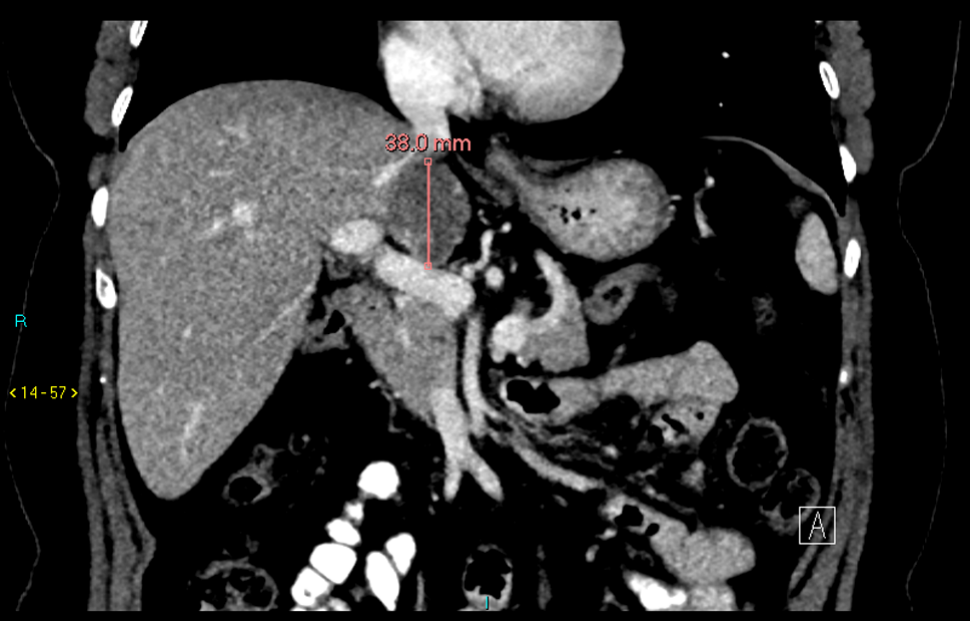
Seven months post-completion SIRT, contrast-enhanced CT showing tumour reduction in size to 38 mm and a volume reduction to 20 cm^3^ and now deemed suitable for surgical resection

Follow-up imaging 2 months after SIRT delivery showed a tumour volume reduction from 350 cm^3^ at baseline to 53 cm^3^. Imaging performed two months later showed there had been further reduction to 30 cm^3^ and finally at 7-month follow-up, a reduction to 20 cm^3^ (Fig. 2). AFP also showed a decrease from > 50,000 at baseline to 759 IU/ML at six months post-SIRT. Due to the good response to SIRT, the patient was surgically re-evaluated and deemed suitable for resection. The tumour had regressed away from the right hepatic vein. As there was still involvement of the IVC, an extended left hepatectomy with IVC replacement was planned. This was to be done with cardiopulmonary bypass to facilitate the replacement of the IVC at the confluence of the hepatic veins.

**Fig. 3:**
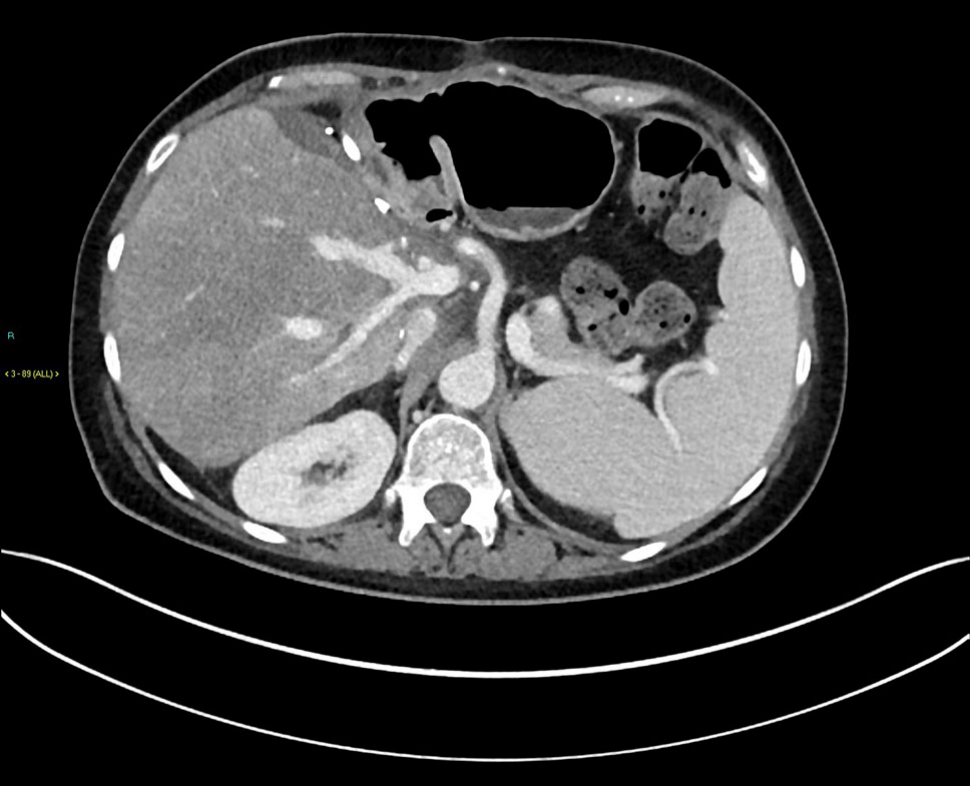
Axial contrast-enhanced CT post-extended left hepatectomy with resection and reconstruction of IVC

An extended left hepatectomy with resection and reconstruction of IVC was performed 8 months post-SIRT (Fig. 3). This required a median sternotomy for cardiopulmonary bypass via cannulas in the aorta, SVC and right atrium. The patient was cooled to 28°, and circulatory arrest was established for the lower half of the body by cross-clamping the descending aorta and IVC. Then, an extended left hepatectomy with resection and reconstruction of the IVC was performed using a Dacron graft.

**Fig. 4:**
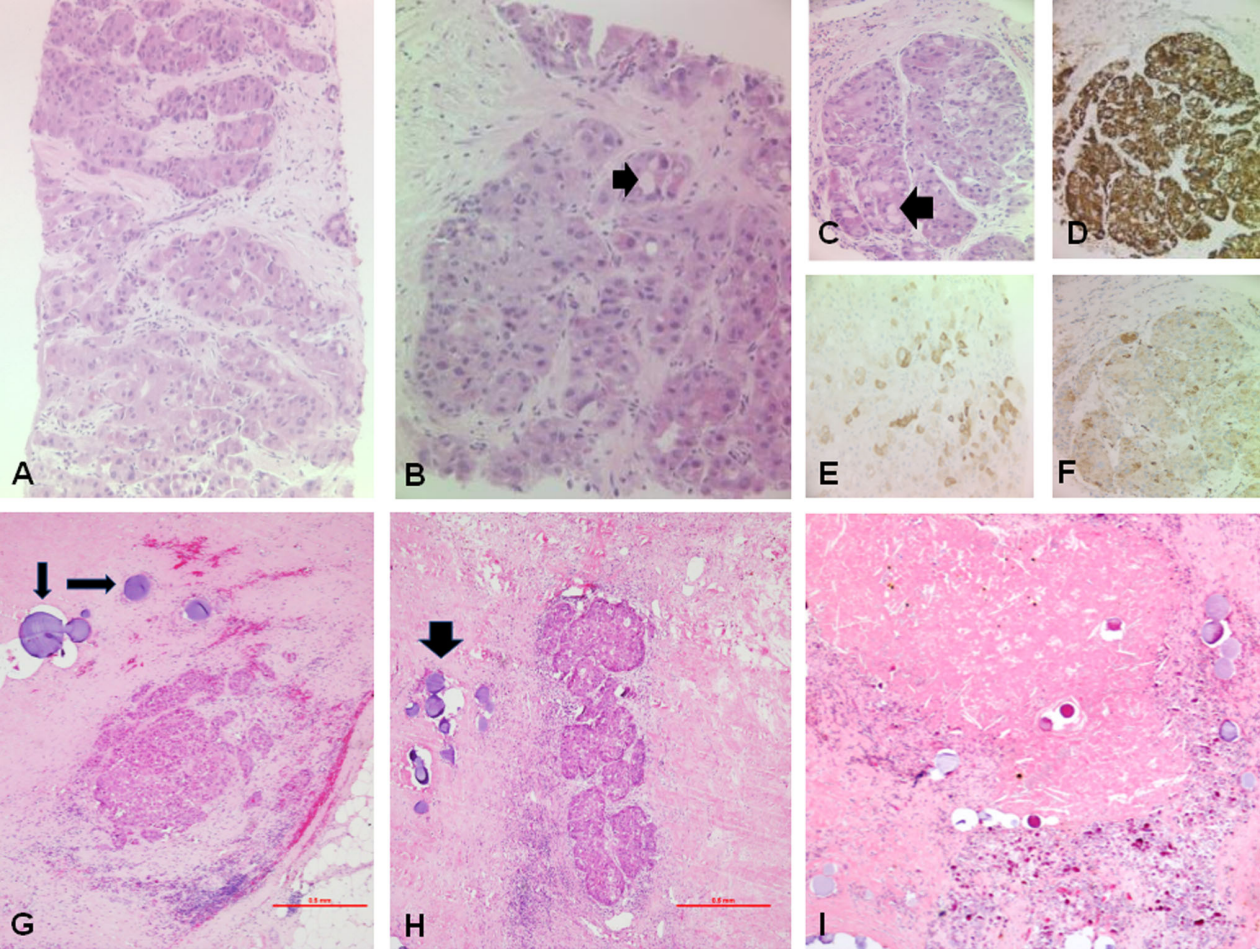
A–F. Liver biopsy of fibrolamellar hepatocellular carcinoma (FL-HCC): **A** Solid nests of large tumour cells with abundant eosinophilic cytoplasm and hyperchromatic or vesicular nuclei and prominent nucleoli are seen within a lamellar fibrous stroma (haematoxylin and eosin—H&E, **A** ×10, **B** ×20, **C** ×20). Intra-cytoplasmic pale bodies (black arrow) are abundant. The FL-HCC cells are typically positive for HepPar1 (**D** ×20), keratin 7 (**E** ×20) and CD68 (**F** ×20). **G**, **I** Resection specimen following TACE and SIRT treatment: **G**, **H** isolated, less than 2-mm sized foci of residual viable FL-HCC (H&E ×4) surrounded by TACE spheres (black arrows). **I** Coagulative tumour necrosis with cholesterol clefts, pigmented macrophages and TACE spheres (H&E ×4)

Histological analysis (Fig. 4) of the resected specimen demonstrated 95% necrosis of the tumour. Only three microscopic foci of residual viable FL-HCC were present (Fig. 4). The resection margin was clear (R0). The background liver was non-cirrhotic with only mild steatosis but no steatohepatitis.

The patient had a post-operative stay of 67 days during which she experienced three grade 3a (Clavien-Dindo) complications including: pleural effusion treated with thoracocentesis, bile leak managed conservatively with percutaneous drainage and partial IVC thrombosis managed with therapeutic anticoagulation. The patient was subsequently discharged with no evidence of recurrence at 2 months of follow-up.

## Discussion

The management of FL-HCC poses several challenges due to its rarity (0.85–16% of all HCCs) and limited treatment options. FL-HCC classically affects younger patients in the absence of chronic liver disease, and unlike HCC AFP levels are typically normal. However, this classic presentation is not always observed with a variation in age at presentation, and in up to 25% of cases AFP can be elevated [11, 12]. Surgical resection is the only proven curative treatment method and reflects the current standard of care [12, 13]. For unresectable FL-HCC, there is no standard of care, and several management strategies have been attempted including chemotherapy, ablation, external beam radiotherapy and intra-arterial treatments [1, 14–17]. Recently, a characteristic DNAJB1–PRKACA fusion transcript has been reported in FL-HCC that could present a therapeutic target in future [18].

Intra-arterial treatment with Y90 with SIRT has not previously been reported for FL-HCC. It is, however, a theoretically attractive treatment option due to its evolving evidence base in traditional HCC and also because FL-HCC may be radiosensitive [5–8, 17, 19]. Y90-loaded microspheres are small (20–60 microns) and deposit distally in the arterial system. As a beta-emitter, the Y90-loaded microspheres have an effective radiotherapy range of about 2.5 mm and induce cell injury through DNA damage [10]. While typically considered a salvage therapy, some patients treated with SIRT can demonstrate a response which renders them surgical candidates. In this case, the FL-HCC showed a tumour volume reduction from 350 cm^3^ at baseline to 20 cm^3^ 7 months post-SIRT. Based on the reduction in size, the tumour was then felt to be surgically resectable but would require partial resection and reconstruction of the IVC. The concept of surgical resection after SIRT is new, and only small case series have shown that it is a technically feasible option in highly selected patients, but never been reported with FL-HCC [20, 21]. Downsizing and subsequent resection has, however, been reported following chemotherapy in the treatment of FL-HCC [22].

Resection after SIRT is associated with a high (42–78%) 90-day morbidity, the most common complication being bile leak [20, 21]. Following resection, three grade 3a or above (Clavien-Dindo) complications were encountered in this case, one of which was also a bile leak but was managed with drainage. The resection was able to achieve a R0 margin and should therefore be regarded as curative.

## Conclusion

FL-HCC is a rare hepatic neoplasm with limited treatment options for unresectable cases. This is the first reported use of SIRT with Y90 in the management of FL-HCC which resulted in significant tumour size reduction allowing the patient to undergo curative surgical resection. While this is an isolated case, SIRT should be a treatment consideration in the multidisciplinary management of unresectable FL-HCC.
